# Fatty Acid Optimization of Locally Produced Ready-to-Use Therapeutic Foods for the Treatment of Acute Malnutrition in Children Using Linear Programming: An Application to India and Pakistan

**DOI:** 10.3390/nu17233653

**Published:** 2025-11-22

**Authors:** Samir Ismail, Amith Umesh, Mohid Khan, Ahmed Moutwakil, Tu Nguyen, Meghana Dantuluri, Akhila Annadanam, Ayush Prasad, Melody Multra, Deepti Sudhakar, Varun Mahadevan, Gul Nawaz Khan, Sajid Soofi, Keith P. West

**Affiliations:** 1 Center for Human Nutrition, Department of International Health, Johns Hopkins Bloomberg School of Public Health, Johns Hopkins University, Baltimore, MD 21218, USA; samirismail08@gmail.com (S.I.); melody.multra@gmail.com (M.M.); sudhakardeepti@gmail.com (D.S.); 2 The Satya Nutrition Foundation, Garland, TX 75040, USA; mohidkhan169@gmail.com (M.K.); amoutwakil@mednet.ucla.edu (A.M.); tunguyen51194@gmail.com (T.N.); mdantuluri@gmail.com (M.D.); vannadanam@gmail.com (A.A.); vmahadevan2017@gmail.com (V.M.); 3 David Geffen School of Medicine, University of California, Los Angeles, CA 90095, USA; 4 Robert Wood Johnson Medical School, Rutgers University, New Brunswick, NJ 08901, USA; 5 Bloomberg School of Public Health, Johns Hopkins University, Baltimore, MD 21205, USA; kwest1@jhu.edu; 6 College of Medicine, University of Cincinnati, Cincinnati, OH 45229, USA; 7 School of Medicine, Creighton University, Omaha, NE 68124, USA; 8 Center of Excellence in Women & Child Health, Aga Khan University, Karachi 74800, Pakistan; gul.nawaz@aku.edu

**Keywords:** RUTF, malnutrition, optimization, neurocognition, linear programming

## Abstract

Background/Objectives: Ready-to-use therapeutic foods (RUTFs) are a common treatment for children under five years diagnosed with acute malnutrition. However, traditional RUTFs are often not locally produced, and the costs of the RUTF can be a barrier to access in India and Pakistan. Our goal was to utilize linear programming (LP) to generate an RUTF formulation based on ingredients locally available in India and Pakistan. We also aim to evaluate the effectiveness of LP in generating such a recipe that is also nutrient-optimized to promote neurocognitive recovery. Methods: The RUTF recipe was generated by applying linear programming to a database of crop ingredients available in India, subject to nutritional constraints. The resulting formulation was produced and evaluated for nutrition content and shelf life. The efficacy of the LP tool was also evaluated based on the formulated product. Results: We demonstrate that the linear programming tool is largely accurate in predicting the true nutritional content of the formulation. Furthermore, the generated formulation, per 100 g, meets many global macronutrient standards for RUTFs while maintaining a predicted cost that is lower than that of industry-standard products. The conducted shelf-life study indicates the viability of the RUTF throughout an accelerated testing period. In addition, the satisfactory consideration of LA and ALA levels provides our RUTF with the potential to address concerns about low DHA levels, and thereby cognitive health, as compared to traditional RUTFs. Conclusions: We use linear programming to generate an affordable and fatty acid-optimized RUTF based on locally available ingredients. Therefore, this formulation holds immense potential to benefit communities in India and Pakistan facing high levels of child malnutrition.

## 1. Introduction

Ready-to-use therapeutic foods (RUTF) are the current gold standard for treating young children ages 6–59 months with moderate to severe acute malnutrition (SAM) [1,2]. Despite their proven effectiveness in supporting physical recovery, there remain challenges in formulating RUTF products that optimally address both nutritional rehabilitation and cognitive development needs [1]. Furthermore, there is a need for more culturally appropriate, sustainable, and economically viable RUTFs in South Asia [3,4]. Current formulations of RUTFs utilize a standard formulation of milk and dairy products, peanuts, vegetable oils, sugar, and a vitamin–mineral premix [1] in various quantities: small (approximately 100–120 kcal/d), medium (approximately 250–499 kcal/d), and large (approximately >500 kcal/d) lipid-based nutrient supplements (LNS) [5] to promote physical recovery.

In this paper, we present a novel RUTF formulation developed using linear programming to optimize nutrient composition while utilizing ingredients available in India and Pakistan. This approach specifically addresses essential nutrient requirements, including essential fatty acids (EFAs) necessary for cognitive development, while considering regional taste preferences, ingredient availability, and cost constraints [3,6]. By leveraging linear programming optimization and locally sourced ingredients, our formulation aims to provide a culturally appropriate and potentially more accessible alternative to imported RUTFs for nutritional rehabilitation of moderately-to-severely malnourished children in South Asia [6].

Malnutrition remains a chronic, underlying cause of preschool childhood mortality, accounting for approximately half of the deaths under the age of 5 years in low- and middle-income countries [7]. Severe acute malnutrition (SAM) in childhood can lead to severe medical complications such as anemia and infections [3,8]. According to UNICEF, 22% of the global pediatric population is stunted (149.2 million children) [4]. Wasting malnutrition is especially prevalent in South Asia, including India and Pakistan [9]. According to the most recent survey data, 34.7% of Indian children and 37.6% of Pakistani children under 5 years of age are stunted [10]. India ranks 105th among 127 countries in the global hunger index, highlighting the severe malnutrition in India despite the country’s rapid economic growth. Similarly, Pakistan ranks 104th among 127 countries in the Global Hunger Index [11]. While SAM has significant impairments on the physical development of a child, it can also have profound impacts on neuropsychiatric and cognitive development [1]. Undernourishment during early childhood has been linked to reduced attention and memory, lower IQ scores, and delayed language acquisition [12]. These deficits can persist into adolescence and adulthood, contributing to poorer academic performance and long-term cognitive disadvantage [12,13].

Ready-to-use therapeutic foods (RUTFs), including lipid-based nutrient supplements (LNSs), represent an efficacious, albeit costly, standard of care for treating and rehabilitating young children with acute moderate-to-severe malnutrition [5,13,14]. Despite these advances, ongoing challenges remain. While current RUTFs and LNS are carefully formulated to provide adequate energy, protein, and micronutrients, there is increasing evidence that some formulations may not optimally address the requirements for essential fatty acids, particularly those critical for cognitive development and recovery [15]. There is insufficient evidence regarding the adequacy of RUTF composition, especially in relation to long-chain polyunsaturated fatty acids such as docosahexaenoic acid (DHA), a key component of neural tissue and a marker for cognitive health. Deficiencies in DHA have been associated with adverse neurodevelopmental outcomes [15,16]. While Omega-3 fatty acid (ALA) is a precursor to DHA, studies have suggested that increasing ALA intake has a negligible effect on increasing plasma DHA level [17]. Previous rodent studies have also indicated that maximizing plasma DHA levels is achieved when ALA and LA constitute approximately 2% of the total fatty acids in the RUTF [18]. Furthermore, although Hsieh et al. [17] observed increased plasma DHA levels in children who were administered RUTFs optimized for the ALA to Omega-6 (LA) ratio in a randomized control trial located in Malawi, there is a critical gap regarding the nutritional and shelf-life validation of a lipid-optimized recipe throughout South Asia.

In addition to addressing cognitive impairment and adhering to nutritional standards in terms of fatty acid content, an issue with currently distributed RUTFs is that they can be costly and inaccessible [19]. Standard RUTF therapy is not widely available in Pakistan or India. In the present case, we use linear programming to specify nutritional constraints of interest to find a recipe that satisfies these constraints at an optimal cost. Although there is a precedent for the use of linear programming in RUTF formulation [20], its application to creating a recipe that addresses required fatty acids in addition to essential macro and micronutrient constraints in South Asia remains limited. Thus, the purpose of this study was to formulate a RUTF that met energy, macronutrient, and essential micronutrient requirements for nutritional recovery, but also optimized ALA:LA content that may help longer-term neurocognitive recovery [17,18]. We have based the recipe on ingredients locally available to minimize costs in India and Pakistan, drawing on tables of Indian food composition, online food availability sites, and local food vendors. We have also attempted to compare cost-effectiveness with established RUTFs with respect to treating acute malnutrition and have simulated the shelf-life of the generated RUTF formulation.

## 2. Materials and Methods

### 2.1. Crop Database

A database of crops available in India and Pakistan and their respective macro/micro-nutrients was compiled using the 2017 Indian Food Composition Table [21]. Approximate average prices of crops were identified from various direct-to-consumer websites (e.g., Amazon and IndiaMART) and were verified with local suppliers in Pakistan and India. The set of ingredients, along with corresponding micro and macronutrients, is available upon request.

### 2.2. Linear Programming

Linear programming (LP) is an optimization technique to maximize or minimize a linear objective function subject to a set of constraints (i.e., macronutrients and micronutrients). Specifically, the objective function sought to minimize the total cost of ingredients used to produce RUTFs while ensuring that these foods met specific nutritional requirements.

LP was conducted in MATLAB R2020A, a programming platform, using the function linprog.m [22], which was implemented in the form [x, fval] = linprog(f,A,b,Aeq,beq,lb,ub,options). The function solves for the minimum price using the price objective function, f, so that the inequality A × x ≦ b is satisfied. Aeq and Beq are equality constraints such that Aeq × x = Beq, with Aeq set as the coefficient of one for each unique ingredient x, and beq as the mass constraint of that ingredient in grams. The A matrix, also known as the coefficient matrix, represents the contribution of each ingredient (decision variable) to each nutritional constraint. Each row of A corresponds to a nutritional requirement (e.g., minimum protein, maximum lipid), and each column corresponds to an ingredient. Thus, an entry of A indicates how much nutrient is contributed by an ingredient. The b matrix (RHS vector) contains the required nutritional target for each constraint in the same order as the rows of A. For lower-bound constraints (e.g., minimum protein), the nutrient coefficients are multiplied by −1, and the corresponding value in b is also negative so that the constraint takes the linear programming standard form of the above inequality. For upper-bound constraints (e.g., maximum total lipid), the nutrient coefficients are included with a positive sign. Together, the pair (A, b) enforces all macronutrient and fatty acid requirements in the model.

The input data for the model consisted of the list of candidate ingredients, their nutrient composition per 100 g (protein, lipid, carbohydrate, α-linolenic acid, linoleic acid, and oleic acid), the price of each ingredient (converted to cost per gram), desired nutrient minimum and maximum requirements as explained below, and a fixed total formulation mass.

The next parameters, lb and ub, define the variables for the lower and upper bounds set on our variables, which were used to alter bounds on targeted ingredients. The last parameter, options, enabled the optimization method to be set to use the dual-simplex algorithm. We used the simplex algorithm to compare results from several optimization programs using the simplex solver.

WHO and UNICEF standards based on Codex Alimentarius guidelines for Ready-To-Use Therapeutic Foods (RUTF) were used to determine the lower bounds and upper bounds for macronutrient, micronutrient, and fatty acid values (Table 1) [23]. Constraints for the various macronutrients detailed in Table 1 were used in the linear programming model—protein, lipid, carbohydrates, and fatty acids, including a-linolenic-acid, linoleic acid, and oleic acid, were required to be met [2]. Micronutrients were excluded from the model, given that these could be supplemented using a premix. Sugar and premix constraints were used in the model, as present in the previous literature and to account for taste and high-quality protein [24].

The upper-bound of the total fatty acid content was experimentally incremented while ensuring the baseline value of ALA and LA of 13% of the total fatty acid content was not exceeded to generate multiple recipes, consistent with the formulation described by Hsieh et al. [17]. Recipes exceeding a 2% constraint were screened out [18,25]. Results of the final recipe were obtained as a list of optimized ingredients with respective ingredient amounts in grams.

### 2.3. Nutritional and Shelf-Life Testing

The formula generated by the LP tool was tested in compliance with the Association of Official Analytical Collaboration (A.O.A.C.) 2023 and Food and Agriculture Organization (FAO) 1992 methods by the Pakistan Council of Scientific & Industrial Research, a government-owned lab located in Lahore, Pakistan. The recipe was analyzed for its moisture and nutritional content, including protein, carbohydrate, lipid, fatty acid content, and micronutrients, as shown in Table 1, under A.O.A.C. 2023 methods for nutritional analysis and micronutrients [26]. Furthermore, an accelerated shelf-life study was conducted at 25 degrees Celsius, selected based on applicability to many regions of South Asia, as well as previous work indicating that the stability found for temperatures of 25–30 degrees Celsius for 12 months would be similar to stability for 6 months at 40 degrees Celsius [27]. The RUTF was analyzed for both sensory and microbiological parameters. Sensory parameters were evaluated on a hedonic scale for the sensory evaluation of foods [28].

## 3. Results

### 3.1. Recipe

Table 2 illustrates the predicted RUTF recipe generated by the LP tool. The recipe includes ingredients that can be locally sourced in India. The nutritional composition of the product (e.g., the weight for laboratory testing) was 100 g, selected based on prior reporting [15].

### 3.2. Linear Programming Tool Efficacy

To evaluate the effectiveness of the linear programming tool, we compared the nutritional composition predicted by the linear programming tool (theoretical) with the laboratory-observed (measured) nutritional composition, as demonstrated in Table 3. The presence of select macronutrients, per 100 g of RUTF product, was calculated for the theoretical and measured formulations. The chosen metric was percent error, $\frac{\left|theoretical\;-\;observed\right|}{theoretical}\;\times \;100,$ as reported in previous investigations evaluating linear programming for RUTF treatments in East Africa [24].

A percent error of 0.75 was found between the true total energy of the RUTF compared to the total energy predicted by the LP tool, and the observed amounts of total energy, carbohydrates, and oleic acid were higher than predicted. The predictions for the macromolecules were each within 10% of the measured values. Of all macronutrients, lipid prediction was most accurate at 3.10%, while prediction of linoleic acid was least accurate, as the amount of LA was predicted to be higher than observed, with a percent error of 66.46%. The amount of ALA was also predicted to be higher than observed, with a percent error of 34.16%.

### 3.3. Nutritional Composition Analysis

To evaluate the nutritional composition of the RUTF product, we compared the presence of select macronutrients in the formula to suggested standards, as presented by the WHO [23]. Table 4 presents the comparisons of the laboratory-measured nutritional composition to the WHO-suggested ranges per 100 g [23]. The presence of proteins, lipids, and fatty acids is indicated as a percentage relative to the total energy of the RUTF product. The quantities of protein, LA, and total energy deviated from the suggested ranges by 13% lower, 19% lower, and 0.46% higher, respectively.

### 3.4. Shelf-Life Study

In addition, the recipe was analyzed for both sensory and microbiological parameters over the course of an Accelerated Stability Test (AST). The predicted shelf life of the RUTF packed in a plastic jar was found to be 1 year at 25 degrees Celsius based on the aforementioned sensory and microbiology parameters.

As shown in Table 5, the sensory evaluation indicated that the RUTF was within *very good* and *good* ranges for all parameters at the start of testing, according to a 1–9 point hedonic test [28], and within the *good* range for all parameters after the AST. As illustrated in Table 4, each of the measured microbiology parameters satisfied the tolerance standards set for commercial RUTFs by the United States Department of Agriculture [19]. For example, the Total Plate Count was within the Standard Plate Count tolerance of 10,000 CFU/g at both the start and end of the testing period. Similarly, Yeast and Mold counts were within tolerable ranges for each microorganism. Further, Staphylococcus aureus, Salmonella, and Escherichia coli were not detected, consistent with the USDA standard.

### 3.5. Cost Analysis

Based on the RUTF composition, the calculated price of the RUTF, including ingredients, shipping, and distribution costs, was $0.21 per serving. The price was calculated based on locally sourced products, including products that can be purchased in bulk if available. As a further analysis of the viability of the RUTF, including price, Table 6 compares the RUTF to Plumpy’Nut^®^, a conventional, industry-standard therapeutic food for the treatment of SAM.

Plumpy’Nut^®^ has been a staple in the treatment of SAM [29], with a nutritional composition satisfying the WHO-suggested values as shown in Table 3 [28]. As illustrated in Table 6, the measured RUTF presents similar macronutrient quantities to Plumpy’Nut^®^, such as protein and lipid amounts. Although measured at 100 g, the presented RUTF product provides more energy (552.52 kcal) in comparison to the 92 g serving size of Plumpy’Nut with corresponding energy of 500 kcal per serving. Further, the cost per serving of the present RUTF at $0.21 is less than the estimated cost per serving of Plumpy’Nut^®^ at $0.30 (per 2022 UNICEF pricing data) [30].

## 4. Discussion

Considering the persistence of acute malnutrition in South Asia, we used an LP tool to formulate a low-cost RUTF with locally grown food ingredients that meet the WHO and UNICEF codex for RUTFs [25]. The LP formulation was based on the nutritional information of the ingredients and their price, as well as standard WHO constraints for RUTFs. Our newly formulated RUTF utilizes ingredients local to India and Pakistan and constrains omega-3 and omega-6 fatty acid content (ALA and LA, respectively) below 2% to optimize for recovery from the neurocognitive detriments of SAM. Additionally, our formulated RUTF is significantly more cost-effective due to its reliance on locally sourced ingredients from India and Pakistan. Our primary aim was to evaluate the ability of LP to predict the nutritional composition of our formulated RUTF based on specific ingredients selected for India. In addition, we aimed to evaluate the shelf-life of the generated recipe.

Previous work in the field indicates the viability of applying linear programming to design RUTFs for use in Africa [6]. For evaluating the accuracy of the LP prediction, Dibari et al. considered energy density to be accurate if the relative difference (e.g., between calculated and laboratory-observed values) was within a 10% threshold and considered protein or lipid difference within <5 g (per 100 g) to be accurate [25]. Dibari et al. observed a relative difference of 3.0%, 17.7%, and -2.9% for energy, protein, and lipid, respectively. Ryan et al. demonstrated that their observed macronutrient content was similar to the calculated content, with most recipe formulations having lipid, protein, and carbohydrate content within 10% relative difference. However, Ryan et al. also measured greater energy in the laboratory-observed RUTF than the LP tool predicted [24]. Our results demonstrate that our LP tool is effective in predicting accurate energy, protein, and lipid values. Protein, lipid, and carbohydrate were each within 10% relative difference (Table 3), and energy was within 1% difference, indicating improvement over the protein prediction of Dibari et al., and the energy content prediction of Ryan et al., The efficacy of the LP tool enables the formulation of a recipe that has amounts of total energy, protein, and lipids fall either within or close to the recommended ranges. For example, the observed total energy is slightly above the suggested range, and the protein content was within 2% of the suggested range. Given the lipid-optimized formula, we note that the ALA levels reside within the suggested range, and the LA levels fall slightly outside the suggested range.

The Accelerated Stability Test (Table 5) illustrates the practical shelf life of the RUTF, as at both the beginning of testing and at the end of the accelerated one-year mark, all microbiology parameters remained within standard, tolerable ranges, with minimal microbial growth. The sensory parameters were also acceptably maintained throughout the accelerated period. In addition, as demonstrated in Table 4, the moisture content is below the recommended 2.5% level, which contributes to minimizing spoilage in RUTFs [28]. The sensory assessment was conducted to confirm product acceptability following accelerated stability testing, but was not performed in the target age group of children aged 1–5 years. Future clinical evaluation will include sensory testing within this population to more accurately assess acceptability and palatability. Furthermore, the testing was performed using reusable glass jars to explore a sustainable packaging option, and the use of these jars may have contributed to a shelf life shorter than the two years recommended by the WHO. Future work will focus on optimizing this packaging approach and extending stability testing to higher temperatures and longer durations, alongside a planned clinical trial to evaluate the formulation’s stability and effectiveness under real-world conditions.

Furthermore, our product provides comparable macronutrient quantities at a 30% lower cost than current industry-leading products [19]. Our product also provides more total energy compared to an industry leader while maintaining a lower cost. Protein sources were identified as significant contributors to the cost. However, using locally sourced ingredients is envisioned to provide lower transportation costs, which can be a significant contributor to total RUTF cost [6]. The use of local ingredients also provides a gateway to sustainability by enabling domestic production of the RUTF. With reduced costs and increased accessibility, our product would be able to provide treatment to a larger population of individuals affected by malnutrition.

While our study offers several advantages, it is important to acknowledge certain limitations. First, the percent error for the prediction of ALA and LA was larger than that of other macro ingredients, which could be due to variations in the source ingredients and reference ingredients, or from minor variations in the manufacturing of the RUTF product. These errors in LA prediction may have contributed to the observed amount of LA falling slightly outside of the WHO-suggested range (Table 4). Low LA levels in nutritional replacement therapy may increase the risk of essential fatty acid deficiency and impaired immune responses in children with severe acute malnutrition. However, such errors can be accounted for after lab testing through manual adjustments to achieve WHO specifications. Further, the errors should not detract from the overall efficacy of the formulation generated by our tool, as a reduction in LA in the actual recipe may aid in cognitive recovery [15]. Second, while our shelf-life study was conducted over a shorter duration than recommended by WHO standards for studies conducted at 25 degrees Celsius [31], the promising results observed for our newly formulated RUTF are highly encouraging. Future studies will include AST at varying temperatures up to 30 degrees. Third, this study evaluated only a single optimized recipe iteration without repeatability testing or alternative formulations. Future work will include validation of the model’s predictive accuracy across multiple recipe iterations and production batches to assess reproducibility and robustness.

In alignment with our fatty acid-optimized RUTF designed for cognitive recovery in children with severe acute malnutrition (SAM), the study by Hsieh et al. demonstrated that RUTFs with an optimized omega-3 to omega-6 fatty acid ratio can effectively increase serum docosahexaenoic acid (DHA) levels in malnourished children, leading to improved cognitive and global development outcomes [17]. In the same study, Hsieh et al. also suggested that lower levels of ALA and LA might stimulate endogenous production of DHA [17]. Our formulation builds upon the work of Hsieh et al. by optimizing the ratio of ALA and LA while also maintaining the total content of ALA and LA below 2% of total fatty acids to maximize DHA. In future investigations, we plan to validate this lipid-optimized formulation with prospective clinical trials to understand its impact on cognitive development in children with SAM in India and Pakistan. We will also investigate the cognitive benefits of adding DHA to our formulation. To further validate the quality of our RUTF, we plan to extend the duration of our shelf-life analysis in future studies.

## 5. Conclusions

This study presents a low-cost RUTF formulation that holds promise for both cognitive and physical recovery in children with SAM. By employing linear programming, we optimized the ratio and amounts of ALA and LA in the RUTF while adhering to WHO standards. Our findings demonstrate the effectiveness of the linear programming tool in generating a nutritious, fatty acid-optimized recipe that can be cost-effective and locally produced in India and Pakistan. Overall, our RUTF formulation offers significant potential in aiding children with SAM in India through a more sustainable and affordable approach that also optimizes for neurocognitive recovery.

## Figures and Tables

**Table 1:** Nutritional constraints used for the Linear Programming tool.

Nutrient	Minimum Value	Maximum Value
Protein	13 (g)	14.75 (g)
Lipid	14.44 (g)	16.39 (g)
Carbohydrate	8.5 (g)	10.13 (g)
Alpha-Linolenic Acid	0 (g)	1.149 (g)
Linoleic Acid	0 (g)	1.149 (g)
Oleic Acid	6.29 (g)	16.39 (g)
Calcium	300 (mg)	
Iron	10 (mg)	
Magnesium	80 (mg)	
Phosphorus	300 (mg)	
Potassium	1100 (mg)	
Sodium	290 (mg)	
Zinc	11 (mg)	
Copper	1.4 (mg)	
Selenium	20 (mcg)	
Vitamin C	50 (mg)	
Thiamin	0.5 (mg)	
Riboflavin	1.6 (mg)	
Niacin	5.0 (mg)	
Pantothenic Acid	3.0 (mg)	
Vitamin B6	0.6 (mg)	
Vitamin B12	1.6 (mcg)	
Folate	200 (mcg)	
Biotin	60 (mcg)	
Vitamin D	17.5 (mcg)	
Vitamin E	20 (mg)	
Vitamin K	22.5 (mcg)	
Vitamin A	2666 (IU)	

**Table 2:** Ingredient quantities for LP-formulated recipes, as pre-cooked estimates.

Food Ingredients	Quantity (g)
Maize	13.68
Milk powder	8.0
Rice flour (brown)	9.02
Soymeal	8.27
Sugar	21.03
Whey isolate	9.0
Palm oil	21
Rapeseed oil	10
Total	100 g

**Table 3:** Comparison of laboratory-observed nutritional composition vs. suggested nutritional composition.

Nutrient	Theoretical (Per 100 g Serving)	Measured (Per 100 g Serving)	Percent Error (%)
Protein	13 (g)	12.02 (g)	7.55
Lipid	34.22 (g)	33.16 (g)	3.10
Carbohydrate	47.09 (g)	51.5 (g)	9.36
Alpha-Linolenic Acid	1.01 (g)	0.66 (g)	34.16
Linoleic Acid	4.45 (g)	1.49 (g)	66.46
Oleic Acid	15.89 (g)	18.9 (g)	18.97
Total Energy	548.38 (kcal)	552.52 (kcal)	0.75

**Table 4:** A comparison of laboratory-observed nutritional composition vs. WHO-suggested nutritional composition of RUTF [23].

Nutrient	Measured Value	WHO Suggested Value
Protein	8.7 (% of total Energy)	10–12 (% of total Energy)
Lipid	54.01 (% of total Energy)	45–60 (% of total Energy)
Alpha-Linolenic Acid	1.08 (% of total Energy)	0.3–2.5 (% of total Energy)
Linoleic Acid	2.43 (% of total Energy)	3–10 (% of total Energy)
Total Energy	552.52 (Kcal/100 g)	520–550 (Kcal/100 g)
Moisture content	1.03 (% Moisture)	<2.5 (% Moisture)

**Table 5:** Sensory parameters (top) resulted from Accelerated Stability Test, on a 1–9 point hedonic scale: *9: very good, 7–8: good, 5–6: average, 1–4: poor. Microbiology parameter analysis (bottom)-CFU/g: Colony Forming Units per gram, MPN: Most Probable Number per gram.*

Sensory Parameters	Fresh/Zero Day	After AST	
Appearance and Color	8	7	
Aroma	9	8	
Texture	8	7	
**Microbiology Parameters**	**Fresh/Zero Day**	**After AST**	**Tolerance**
Total Plate Count (CFU/g)	1.2 × 10^2^	1.8 × 10^2^	10,000
Total coliforms (MPN/g)	Not detected	Not detected	3
*E. coli* (MPN/g)	Not detected	Not detected	Not detected/Negative
*Salmonella* ssp./25 g	Not detected	Not detected	Not detected/Negative
*Staph. aureus*/g	Not detected	Not detected	Not detected/Negative
Yeast and Mold count (CFU/g)	≤10	≤10	Yeast ≤ 10 Mold ≤ 10

**Table 6:** Comparison of key laboratory-observed quantities vs. industry standard product, based on nutritional and pricing data available for the industry standard product in 2022.

Quantity Per Serving	Measured RUTF (100 g)	Plumpy’Nut^®^ (92 g)
Protein (g)	12.02	12.8
Lipid (g)	33.16	30.3
Energy (kcal)	552.52	500
Price (USD)	$0.21	$0.30

## Data Availability

The data presented in this study are available on request from the corresponding author due to privacy.

## Abbreviations

The following abbreviations are used in this manuscript:

ALA	Alpha-linolenic acid
AST	Accelerated stability test
DHA	Docosahexaenoic acid
LA	Linoleic acid
LP	Linear programming
RUTF	Ready-to-use therapeutic food
PUFA	Polyunsaturated fatty acids
SAM	Severe acute malnutrition

## References

[1] Brenna J.T., Akomo P., Bahwere P., Berkley J.A., Calder P.C., Jones K.D., Liu L., Manary M., Trehan I., Briend A. (2015). Balancing omega-6 and omega-3 fatty acids in ready-to-use therapeutic foods (RUTF). BMC Med.

[2] Schoonees A., Lombard M.J., Musekiwa A., Nel E., Volmink J. (2019). Ready-to-use therapeutic food (RUTF) for home-based nutritional rehabilitation of severe acute malnutrition in children from six months to five years of age. Cochrane Database Syst. Rev..

[3] Chatterjee P. (2021). India’s child malnutrition story worsens. Lancet Child. Adolesc. Health.

[4] FAO, IFAD, UNICEF, WFP, WHO (2021). The State of Food Security and Nutrition in the World 2021. http://www.fao.org/documents/card/en/c/cb4474en.

[5] Dewey K.G., Arnold C.D., Wessells K.R., Stewart C.P. (2023). Lipid-based nutrient supplements for prevention of child undernutrition: When less may be more. Am. J. Clin. Nutr..

[6] Dibari F., Diop E.H.I., Collins S., Seal A. (2012). Low-cost, ready-to-use therapeutic foods can be designed using locally available commodities with the aid of linear programming. J. Nutr..

[7] Azevedo J.P., Banerjee A., Wilmoth J., Fu H., You D. (2024). Hard truths about under-5 mortality: Call for urgent global action. Lancet.

[8] Caulfield L.E., Richard S.A., Rivera J.A., Musgrove P., Black R.E., Jamison D.T., Breman J.G., Measham A.R., Alleyne G., Claeson M., Evans D.B., Jha P., Mills A., Musgrove P. (2006). Stunting, Wasting, and Micronutrient Deficiency Disorders. Disease Control Priorities in Developing Countries.

[9] Webb P., Stordalen G.A., Singh S., Wijesinha-Bettoni R., Shetty P., Lartey A. (2018). Hunger and malnutrition in the 21st century. BMJ.

[10] Global Nutrition Report Country Nutrition Profiles—Global Nutrition Report. https://globalnutritionreport.org/resources/nutrition-profiles/asia/southern-asia/india/.

[11] Concern Worldwide, Welthungerhilfe, Institute for International Law of Peace and Armed Conflict (IFHV) 2024 Global Hunger Index. https://www.globalhungerindex.org/ranking.html.

[12] Mwene-Batu P., Bisimwa G., Baguma M., Chabwine J., Bapolisi A., Chimanuka C., Molima C., Dramaix M., Kashama N., Macq J. (2020). Long-term effects of severe acute malnutrition during childhood on adult cognitive, academic and behavioural development in African fragile countries: The Lwiro cohort study in Democratic Republic of the Congo. PLoS ONE.

[13] Dewey K.G., Arnold C.D., Wessells K.R., Prado E.L., Abbeddou S., Adu-Afarwuah S., Ali H., Arnold B.F., Ashorn P., Ashorn U. (2022). Preventive small-quantity lipid-based nutrient supplements reduce severe wasting and severe stunting among young children: An individual participant data meta-analysis of randomized controlled trials. Am. J. Clin. Nutr..

[14] Potani I., Spiegel-Feld C., Brixi G., Bendabenda J., Siegfried N., Bandsma R.H.J., Briend A., Daniel A.I. (2021). Ready-to-Use Therapeutic Food (RUTF) Containing Low or No Dairy Compared to Standard RUTF for Children with Severe Acute Malnutrition: A Systematic Review and Meta-Analysis. Adv. Nutr..

[15] Stephenson K., Callaghan-Gillespie M., Maleta K., Nkhoma M., George M., Park H.G., Lee R., Humphries-Cuff I., Lacombe R.S., Wegner D.R. (2022). Low linoleic acid foods with added DHA given to Malawian children with severe acute malnutrition improve cognition: A randomized, triple-blinded, controlled clinical trial. Am. J. Clin. Nutr..

[16] Weiser M.J., Butt C.M., Mohajeri M.H. (2016). Docosahexaenoic Acid and Cognition throughout the Lifespan. Nutrients.

[17] Hsieh J.-C., Liu L., Zeilani M., Ickes S., Trehan I., Maleta K., Craig C., Thakwalakwa C., Singh L., Brenna J.T. (2015). High-Oleic Ready-to-Use Therapeutic Food Maintains Docosahexaenoic Acid Status in Severe Malnutrition. J. Pediatr. Gastroenterol. Nutr..

[18] Gibson R.A., Neumann M.A., Lien E.L., Boyd K.A., Tu W.C. (2013). Docosahexaenoic acid synthesis from alpha-linolenic acid is inhibited by diets high in polyunsaturated fatty acids. Prostaglandins Leukot. Essent. Fat. Acids.

[19] UNICEF (2023). Ready-to-Use Therapeutic Food: Market and Supply Update. https://www.unicef.org/supply/reports/ready-use-therapeutic-food-market-and-supply-update.

[20] Sheibani E., Dabbagh Moghaddam A., Sharifan A., Afshari Z. (2018). Linear programming: An alternative approach for developing formulations for emergency food products. J. Sci. Food Agric..

[21] Longvah T., An̲antan̲ I., Bhaskarachary K., Venkaiah K. (2017). Indian Food Composition Tables.

[22] Linprog—Solve Linear Programming Problems. https://www.mathworks.com/help/optim/ug/linprog.html.

[23] World Health Organization (2021). WHO Guideline on the Dairy Protein Content in Ready-to-Use Therapeutic Foods for Treatment of Uncomplicated Severe Acute Malnutrition.

[24] Ryan K.N., Adams K.P., Vosti S.A., Ordiz M.I., Cimo E.D., Manary M.J. (2014). A comprehensive linear programming tool to optimize formulations of ready-to-use therapeutic foods: An application to Ethiopia. Am. J. Clin. Nutr..

[25] Jones K.D.J., Ali R., Khasira M.A., Odera D., West A.L., Koster G., Akomo P., Talbert A.W.A., Goss V.M., Ngari M. (2015). Ready-to-use therapeutic food with elevated n-3 polyunsaturated fatty acid content, with or without fish oil, to treat severe acute malnutrition: A randomized controlled trial. BMC Med..

[26] (1995). Official Methods of Analysis of AOAC International.

[27] Zuzarte A., Mui M., Ordiz M.I., Weber J., Ryan K., Manary M.J. (2020). Reducing Oil Separation in Ready-to-Use Therapeutic Food. Foods.

[28] Hadi S., Amani R., Tehrani M.M., Hadi V., Hejri S., Askari G. (2022). Ready-to-Use Therapeutic Food (RUTF) Formulations with Functional Food and Nutrient Density for the Treatment of Malnutrition in Crisis. Int. J. Prev. Med..

[29] Enserink M. (2008). The Peanut Butter Debate. Science.

[30] UNICEF Ready-to-Use Therapeutic Food (RUTF) Price Data. https://www.unicef.org/supply/media/24306/file/Ready-to-use-therapeutic-food-price-2003-2024.pdf.

[31] James G., Stephenson K., Callaghan-Gillespie M., Kamara M.T., Park H.G., Brenna J.T., Manary M.J. (2023). Docosahexaenoic Acid Stability in Ready-to-Use Therapeutic Food. Foods.

